# ENHANCING WELL-BEING THROUGH EXERCISE DURING OLDER AGE

**DOI:** 10.1093/geroni/igac059.1552

**Published:** 2022-12-20

**Authors:** David Marquez

**Affiliations:** University of Illinois Chicago, Chicago, Illinois, United States

## Abstract

Dr. Marquez’s Roybal pilot Enhancing Well-Being Through Exercise During Older Age piloted an adaptation of a group exercise evidence-based program for people with arthritis symptoms, Fit & Strong!, that included education on emotional wellbeing and mental health. The pilot used a waitlist design to test the 8-week program among Black residents reporting depressive symptoms living in a senior housing facility. A battery of physical, emotional, and cognitive outcomes were collected pre and post intervention and a subset of participants (n=11) received MRIs pre and post. The sample included 28 participants (n=13 intervention, n=15 waitlist) who had a mean age of 69.8, were 89% female, reported a mean of 3.6 chronic conditions, and an average WOMAC pain score of 5.7 (moderate pain). Though under-powered, analyses show greater improvements in key physical activity and cognitive domains among the intervention group compared to the waitlist group.

